# Pulmonary Intravascular Large B‐Cell Lymphoma Diagnosed by Transbronchial Lung Cryobiopsy: A Case Report

**DOI:** 10.1002/rcr2.70226

**Published:** 2025-06-16

**Authors:** Yen Shen Wong, Hideaki Furuse, Yuji Matsumoto, Kenya Sato, Takaaki Tsuchida

**Affiliations:** ^1^ Department of Endoscopy, Respiratory Endoscopy Division National Cancer Center Hospital Tokyo Japan; ^2^ Faculty of Medicine Universiti Teknologi MARA Sungai Buloh Selangor Malaysia; ^3^ Department of Thoracic Oncology National Cancer Center Hospital Tokyo Japan

**Keywords:** bronchoscopy, pulmonary intravascular large B‐cell lymphoma, transbronchial lung cryobiopsy

## Abstract

Intravascular large B‐cell lymphoma (IVLBCL) is a rare and aggressive extranodal lymphoma characterised by the proliferation of malignant lymphocytes within the lumens of blood vessels. Diagnosing IVLBCL is often challenging due to its nonspecific clinical presentation and difficulty in obtaining histopathologic confirmation. Here, we present a case of IVLBCL diagnosed by transbronchial lung cryobiopsy (TBLC). A 68‐year‐old man was initially diagnosed with diffuse large B‐cell lymphoma based on an orchiectomy specimen. Although initial treatment achieved complete remission, tumour marker levels progressively increased during follow‐up. TBLC was performed after bone marrow biopsy, cerebrospinal fluid cytology, and a random skin biopsy failed to detect lymphoma lesions. Histological findings of the TBLC specimen demonstrated medium‐sized atypical lymphocytes filling the blood vessels, leading to a diagnosis of IVLBCL. Cryobiopsy specimens allow for the evaluation of alveolar structure and microvasculature, making it a valuable and less invasive diagnostic option for pulmonary IVLBCL.

## Graphical abstract

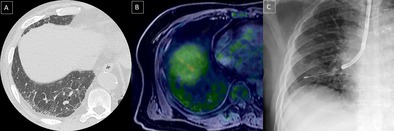

## 1 Introduction

Intravascular large B‐cell lymphoma (IVLBCL) represents a rare and distinctive variant of non‐Hodgkin lymphoma characterised by the selective proliferation of neoplastic lymphoid cells within blood vessel lumina [1]. World Health Organisation defines IVLBCL as a distinct extranodal B‐cell lymphoma marked by the selective growth of neoplastic lymphoid cells within blood vessel lumina, particularly within capillaries [2]. These malignant cells can infiltrate the microvasculature of any organ, causing occlusion and diverse clinical manifestations, often delaying diagnosis and worsening prognosis. In this context, we present a case demonstrating the utility of transbronchial lung cryobiopsy (TBLC) in diagnosing pulmonary IVLBCL.

## 2 Case Report

**FIGURE 1:**
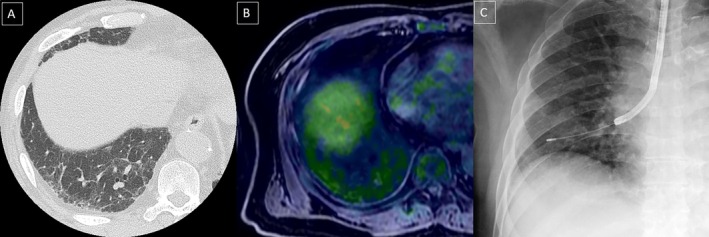
Imaging findings of the patient. (A) High‐resolution computed tomography of the chest showing reticular shadows and ground‐glass opacities in the lower lobe. (B) Positron emission tomography–magnetic resonance imaging demonstrating faint fluorodeoxyglucose uptake in the lower lobe. (C) Transbronchial lung cryobiopsy performed in the subpleural area of the right lower lobe.

A 68‐year‐old man with right testicular swelling had undergone orchiectomy and was diagnosed with diffuse large B‐cell lymphoma (DLBCL) 2 years ago. He received six cycles of R‐CHOP chemotherapy, followed by four cycles of prophylactic intrathecal methotrexate. As a result of the treatment, the DLBCL initially achieved complete remission based on ^18^F‐fluorodeoxyglucose‐positron emission tomography (FDG‐PET). However, during follow‐up, he complained of dizziness, and his soluble interleukin‐2 receptor (sIL‐2R) levels progressively increased from 747 to 8622 U/mL in 12 months (Table 1). Whole‐body contrast‐enhanced computed tomography (CT) scan showed no tumour recurrence, except for reticular shadows and ground‐glass opacities in the lower lobes of both lungs, which were suggestive of interstitial pneumonia. Although PET‐magnetic resonance imaging (MRI) also showed no tumour recurrence, there was faint FDG uptake in both lower lungs (Figure 1). Bone marrow aspiration and biopsy revealed no lymphoma infiltration, and cerebrospinal fluid (CSF) cytology showed no malignant cells. Multiple random skin biopsies also failed to show any lymphoma lesions.

**TABLE 1:** Laboratory data of the patient before the procedure.

Parameters	Results	Unit	Parameters	Results	Unit
WBC	8.5	10^3^/μL	TP	6.7	g/dL
Neu	5.87	%	Alb	3.1	g/dL
Lym	1.11	%	T‐Bil	0.9	mg/dL
Mono	1.28	%	AST	33	U/L
Eos	2.0	%	ALT	15	U/L
RBC	340	10^4^/μL	ALP	303	U/L
Hb	10.3	g/dL	LDH	550	U/L
Hct	30.3	%	BUN	15	mg/dL
PLT	6.7	10^4^/μL	Na	138	mmol/L
sIL‐2R	8622	U/mL	K	3.8	mmol/L
PT‐INR	1.25		Ca	8.6	mg/dL
APTT	29.4	s			

Abbreviations: Alb, albumin; ALP, alkaline phosphatase; ALT, alanine transaminase; APTT, activated partial thromboplastin time; AST, aspartate transaminase; BUN, blood urea nitrogen; Ca, calcium; Eos, eosinophils; Hb, haemoglobin; Hct, haematocrit; K, potassium; LDH, lactate dehydrogenase; Lym, lymphocytes; Mono, monocytes; Na, sodium; Neu, neutrophils; PLT, platelet; PT‐INR, prothrombin time‐international normalised ratio; RBC, red blood cell; sIL‐2R, soluble interleukin‐2 receptor; T‐Bil, total bilirubin; TP, total protein; WBC, white blood cell.

Due to the inconclusive diagnostic workup, TBLC was scheduled for the lung lesion. A platelet transfusion was administered prior to the procedure for thrombocytopenia (Table 1). Under direct bronchoscopic visualisation (BF‐1T260, Olympus, Tokyo, Japan), a 6‐Fr balloon catheter (E‐080‐6F, Edwards Lifesciences, Irvine, CA) was inserted into the right lower lobe bronchus [3]. After securing airway access with an 8.0‐mm endobronchial tube, a 1.9‐mm reusable cryoprobe (20402‐040, Erbe Elektromedizin GmbH, Tübingen, Germany) was advanced into the right lower bronchus. To secure diagnostic sensitivity, four sequential TBLCs were performed from multiple subpleural sites in the right lower lobe. Haemorrhage control was achieved by inflation of the endobronchial balloon, resulting in only mild (Grade 1) bleeding.

**FIGURE 2:**
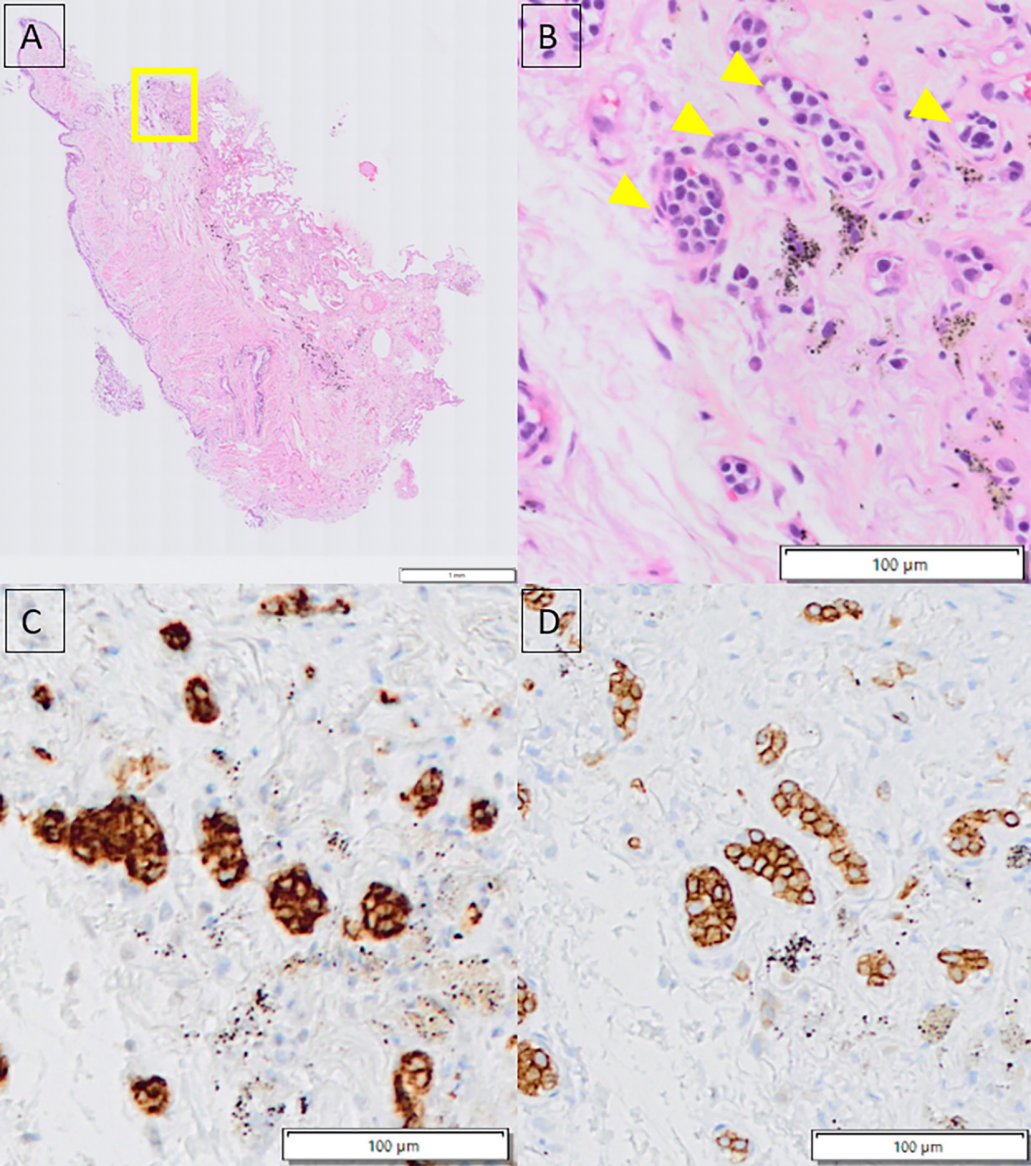
Histological findings of a lung cryobiopsy specimen. (A) A cryobiopsy sample measuring 4.6 × 4.2 mm^2^ demonstrating well‐preserved tissue architecture without crush artefact. (B) At higher magnification (square), haematoxylin and eosin staining reveal atypical lymphoma cells confined within capillary lumina (arrowheads), a hallmark of intravascular large B‐cell lymphoma. (C, D) Immunohistochemical staining shows neoplastic cells positive for CD20 and CD79a respectively, confirming the B‐cell lineage of the malignant cells.

One of the four specimens obtained was diagnostic, measuring approximately 4.6 × 4.2 mm^2^. Histological findings of the TBLC specimen demonstrated medium‐sized atypical lymphocytes filling the blood vessels. Immunohistochemical staining showed positivity for CD20, CD79a and PAX5, leading to the diagnosis of IVLBCL (Figure 2). The patient subsequently received three cycles of T‐ESHAP chemotherapy. His sIL‐2R levels significantly improved following the treatment.

## 3 Discussion

In our case, the specimen obtained via TBLC led to a definitive diagnosis of IVLBCL, allowing the patient to receive appropriate next‐step treatment.

IVLBCL traditionally manifests with central nervous system, cutaneous, or bone marrow involvement, where diagnosis is typically achieved through CSF analysis, skin biopsy or bone marrow aspiration [4]. Despite the patient's history of testicular DLBCL and rising sIL2‐R levels, initial diagnostic efforts including bone marrow examination, CSF analysis, and multiple random skin biopsies were non‐diagnostic. This comprehensive negative workup led to the consideration of alternative diagnostic approaches.

CT scans typically show bilateral ground‐glass opacities with reticulations in 91% of IVLBCL cases, reflecting expanded capillaries filled with neoplastic lymphoid cells [5]. FDG‐PET findings can be subtle and mimic inflammation. In our case, PET‐MRI showed faint FDG uptake in both lungs, initially misinterpreted as interstitial pneumonia. This highlights the need for further investigation when clinical suspicion is high despite mild FDG uptake.

The use of TBLC represents a significant advancement in the diagnostic approach to suspected pulmonary IVLBCL. Conventional forceps biopsy often causes crush artefacts and cannot preserve the vascular architecture necessary for the definitive diagnosis of intravascular pathologies [6]. TBLC has emerged as a valuable diagnostic tool, with recent systematic reviews demonstrating its utility as an alternative to video‐assisted thoracoscopic surgery (VATS) in interstitial lung disease diagnosis [7]. A comprehensive analysis of studies from 2010 to 2020 revealed that TBLC achieved a pooled diagnostic yield of 76.8%, increasing to 80.7% in centres with greater procedural experience [8]. This technique offers several advantages for IVLBCL diagnosis, enabling the collection of larger tissue samples with well‐preserved vascular architecture [9]. This preservation of tissue architecture is crucial for visualising lymphoma cells within vessel lumina and facilitating accurate immunohistochemical analysis. Furthermore, TBLC demonstrates an acceptable safety profile with manageable complication rates: pneumothorax requiring chest tube in 5.6% and a mortality rate of 0.6%, which compares favourably to the 1.7% mortality rate associated with VATS [8].

TBLC has a higher bleeding risk than traditional biopsy techniques due to the freezing process, which causes extensive tissue damage and capillary disruption [10]. A meta‐analysis reported a significant bleeding rate of 9.9% for TBLC procedures [8]. Additionally, the patient had thrombocytopenia, which requires us to exercise extreme caution regarding bleeding. We adopted a modified balloon occlusion method with a 6‐Fr balloon catheter to control the bleeding [3]. As a result, we successfully achieved adequate haemostasis with only Grade 1 bleeding despite multiple biopsies. This method provides high stability and a reliable tamponade effect, potentially strengthening the safety profile of this diagnostic procedure.

In summary, we present a case of pulmonary IVLBCL diagnosed by TBLC. Cryobiopsy is a less invasive and effective diagnostic technique that offers superior tissue preservation and aids in the early detection of this rare lymphoma variant.

## Author Contributions

All listed authors contributed to the article.

## Ethics Statement

The authors declare that written informed consent was obtained for the publication of this manuscript and accompanying images using the consent form provided by the Journal.

## Conflicts of Interest

The authors declare no conflicts of interest.

## Data Availability

The data that support the findings of this study are available from the corresponding author upon reasonable request.
